# Making a meaningful impact: modelling simultaneous frictional collisions in spatial multibody systems

**DOI:** 10.1098/rspa.2014.0859

**Published:** 2015-05-08

**Authors:** Thomas K. Uchida, Michael A. Sherman, Scott L. Delp

**Affiliations:** 1 Department of Bioengineering, Stanford University, 318 Campus Drive West, James H. Clark Center, Stanford, CA 94305-5448, USA; 2 Departments of Mechanical Engineering and Orthopaedic Surgery, Stanford University, 318 Campus Drive West, James H. Clark Center, Stanford, CA 94305-5444, USA

**Keywords:** collision, contact, impact, multibody dynamics, non-smooth dynamics

## Abstract

Impacts are instantaneous, computationally efficient approximations of collisions. Current impact models sacrifice important physical principles to achieve that efficiency, yielding qualitative and quantitative errors when applied to simultaneous impacts in spatial multibody systems. We present a new impact model that produces behaviour similar to that of a detailed compliant contact model, while retaining the efficiency of an instantaneous method. In our model, time and configuration are fixed, but the impact is resolved into distinct compression and expansion phases, themselves comprising sliding and rolling intervals. A constrained optimization problem is solved for each interval to compute incremental impulses while respecting physical laws and principles of contact mechanics. We present the mathematical model, algorithms for its practical implementation, and examples that demonstrate its effectiveness. In collisions involving materials of various stiffnesses, our model can be more than 20 times faster than integrating through the collision using a compliant contact model. This work extends the use of instantaneous impact models to scientific and engineering applications with strict accuracy requirements, where compliant contact models would otherwise be required. An open-source implementation is available in Simbody, a C++ multibody dynamics library widely used in biomechanical and robotic applications.

When bodies collide, destruction aside, the typical outcomes are two:

If contacts persist, constraints then exist, but impulses also accrue.

The work done by friction (that's sliding, not stiction) makes energy wane and subdue;

Throughout the colliding, the points will stop sliding, and sticking will surely ensue.

We compress and expand on one time-constant hand; velocities change, we've decreed –

With impact performed, but nothing deformed, the time-stepper then can proceed.

Thomas K. Uchida

## 1. Collisions in multibody systems

Collisions are short-duration, pairwise interactions during which contact forces change rapidly. Given a high-fidelity contact model, a collision may be simulated accurately by repeatedly evaluating realistic instantaneous contact forces while integrating with very small timesteps. *Compliant contact models* use finite elements or analytical methods to include material compliance, dissipation and friction effects, and can provide accurate, time-resolved information about the interaction between bodies during a collision [1]. Although time-resolved information about a collision is useful in some contexts, we are often uninterested in these details and are unwilling or unable to invest the computational resources required to calculate them. We must still accurately determine the macroscale effect of each collision on the simulation trajectory, but would gladly forgo the microscale details to improve computation speed.

*Rigid contact models* address this need by modelling each collision as an instantaneous impact event where no simulation time elapses and no configuration changes occur between the beginning and end of the impact, and yielding only the accumulated impulse rather than a time history of contact forces. Persisting contact following a collision is then simulated using algebraic constraints, which is a more efficient strategy than using a compliant contact model to repeatedly evaluate instantaneous contact forces. Intermittent contact is commonly encountered in biomechanical and robotic simulations, such as those involving gait and manipulation tasks; an impact model is an essential component of an efficient simulation strategy for these applications.

Simplified impact models have been developed for physics-based games and real-time simulators, but physical laws are often relaxed or ignored in favour of satisfying real-time performance constraints, which are more important in these applications. For example, a polyhedral approximation of the friction cone is commonly employed to facilitate forming a linear complementarity problem [2–4], which can be solved efficiently, but non-physical, coordinate frame–dependent, anisotropic friction behaviour is introduced. Newton's velocity-based restitution model is also used despite its well-known potential to produce energetically inconsistent results when applied in complex contact situations [5–9]. Another common simplification is to ignore the internal structure of the impact process and assume sliding directions remain constant [10,11], which can lead to large macroscale errors, as we will show. Other existing methods focus on efficiently solving large-scale problems, such as those involving granular media, and adopt many similar simplifications. For example, Smith *et al.* [11] ignore the coupling between normal and tangential impulses, assume a single coefficient of restitution (COR) for all simultaneous impacts, disregard changes in sliding direction during a collision, and do not attempt to find a unique solution in the presence of redundant constraints, yet they obtain good results for granular media. In many cases, these simplifications are appropriate for the problems at hand; we will argue that they are not appropriate for the problems discussed here. We refer the interested reader to the review paper by Gilardi & Sharf [12] and the more recent paper by Khulief [13] for a thorough survey of impact modelling.

Our objective in this work is to simulate intermittent contact in biomechanical and robotic applications that demand realistic results, but where a compliant contact model would lead to intolerably slow simulations. Noteworthy examples are the development of gait controllers [14,15] and predictive simulations of motion [16], both of which can be used to understand human mobility and how movement changes in response to fatigue, injury, ageing and assistive device augmentation. These simulation paradigms demand efficient yet accurate models to arrive at optimization problems that are tractable yet produce realistic results. We view our rigid model as an approximation of our compliant model [17] and, thus, deem comparison with the compliant model to be the appropriate metric for evaluation; experimental validation is not the goal of this model reduction procedure.

Modelling a collision as an instantaneous event devoid of configuration changes is but an approximation of reality; however, we need not forsake all physical laws [11]. For example, the law of conservation of energy, Newton's laws of motion, and coordinate frame invariance ought to hold in every physics-based simulation. During impact, normal impulses should be strictly repulsive, friction impulses should be maximally dissipative, and the total impulse at each point should lie on or within a smooth friction cone following Coulomb's law. Qualitatively, simulation behaviour should be insensitive to small perturbations that are within numerical uncertainties. We also expect impulses to be distributed among all redundant constraints, not to an effectively random subset as is typical in practice. This mandate ensures we preserve the load-spreading effect exhibited by compliant materials in contact. Of course, to be useful, an impact model must embody these characteristics while being computationally efficient and insensitive to the numerical approximation and round-off errors that affect all numerical simulations. In this paper, we describe a new impact model, called PLUS, that satisfies the criteria discussed above and those listed explicitly in §2a.

The PLUS acronym reflects a pronounceable subset of the people whose work most influenced the algorithm (Poisson, Lankarani, Uchida, Sherman). PLUS uses Poisson's coefficient of restitution [18,19], was inspired by the work of Lankarani [20], and was developed by the present authors. The PLUS model is unique in that it approximates the behaviour of a compliant contact model, adheres to a broad set of physical laws and principles of contact mechanics, considers numerical tolerances explicitly, and does so with modest computational expense. Despite occurring instantaneously in a simulation, each impact is modelled as a compression phase followed by an expansion phase (following Poisson), where the COR is velocity dependent, as suggested experimentally. Each phase is divided into intervals of sliding and rolling (following Lankarani), where sliding and rolling points have, respectively, non-negligible and negligible relative velocities in the tangent plane. The sequence of intervals comprising an impact forms a piecewise linear approximation of a nonlinear phenomenon. Because time does not advance, we employ the Darboux–Keller method [21,22] of tracking the evolution of each impact process using the accumulated normal impulse, which is initially zero and increases monotonically. This approach is similar to that of Mirtich & Canny [23], who compute post-impact velocities by integrating over the normal impulse. Lankarani adopted Routh's graphical method of tracking the evolution of an impact process [24], echoing the work of Wang & Mason [6] and extending the work of Ahmed *et al.* [25].

Lankarani defined seven distinct impact types for strictly sequential point-on-plane collisions in planar multibody systems. In this work, we consider the more general case of simultaneous impacts in spatial multibody systems modelled with generalized coordinates and containing arbitrary holonomic and non-holonomic constraints. We stress the importance of treating impacts as spatial phenomena, since collisions in spatial multibody systems do not remain planar in general. As will be shown, sliding velocities can undergo substantial changes in direction over the duration of a collision. We also emphasize that simultaneity is essential for preserving symmetry where symmetry is expected [11], and demands explicit consideration of numerical tolerances.

We are interested in applications of moderate size involving dozens of rigid bodies, not large-scale problems such as those involving granular flows [11,26] where precise treatment of individual collisions is prohibitively expensive. Consequently, we present the PLUS formulation under the assumption that it is invoked by a separate time integration scheme that has detected a collision and has isolated it in time and configuration to within an acceptable tolerance. It is also possible to combine low-order velocity-level timestepping and impact handling [4,27]; PLUS can be used in such a context, but that discussion lies beyond the scope of this paper. We focus here on the *impact event handler*, the algorithm that computes the impulsive forces and the points at which they must be applied to colliding bodies to prevent interpenetration, obey friction laws and dissipate energy appropriately. The resulting discontinuous velocity changes must obey all constraints. These new velocities are then used as initial conditions when time integration resumes following the collision.

The impact event handler is generally only one of three related components, the others being the collision detection algorithm and the contact handler. The collision detection algorithm is responsible for determining when a collision occurs and, if necessary, isolating the event in time and space to within specified tolerances. The contact handler determines the non-impulsive forces produced by persisting contacts. Neither the collision detection algorithm nor the contact handler will be discussed here.

The remainder of the paper is organized as follows. In §2, we introduce the PLUS model in the context of a single-point-on-plane impact. We state the governing equations alongside the physical laws they represent, and discuss the solution of the resulting systems of equations. The PLUS model is generalized to simultaneous impacts in §3, and we present an algorithm for processing impacts in the presence of redundant constraints. We also address the handling of impacts induced by collisions occurring elsewhere in the system. In §4, we present four examples to demonstrate the features of our model and compare its performance with that of our compliant contact model. The characteristics of the PLUS impact model are summarized in §5, and directions for future work are discussed.

## 2. The PLUS model. Part I: single-point impacts

**Figure 1.**
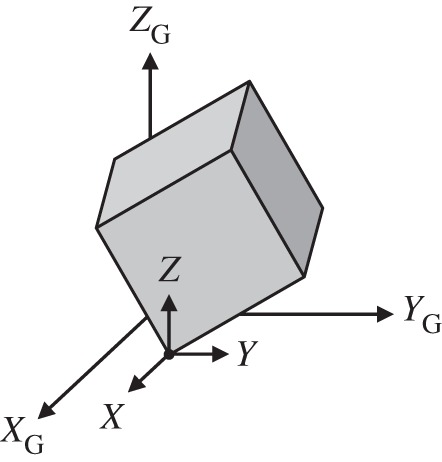
A single vertex of a brick impacts a horizontal ground plane G spanned by the *X*_G_ and *Y*_G_ axes. Impulses are applied to the impacting point to prevent interpenetration, obey friction laws and dissipate energy appropriately. Tangential impulses are applied in the *X*- and *Y*-directions; the normal impulse is applied in the *Z*-direction.

The PLUS impact model approximates the behaviour of a compliant contact model while respecting basic physical laws and preserving fundamental principles of contact mechanics—to the extent possible within a limited computational budget. In this section, we summarize the principles incorporated into the model, present the equations governing the dynamics of an isolated impact, discuss the physical laws these equations represent, and describe how this approach captures the salient aspects of an impact process. PLUS can be used to simulate a variety of unilateral constraints, such as joint stops and ratchets. For clarity, however, we limit our discussion to an impact between a single vertex of a brick and a horizontal ground plane (figure 1), and generalize this theory to simultaneous impacts in §3.

### (a) Concepts incorporated into the PLUS model

The following physical laws, mechanical principles and empirical observations are incorporated into the PLUS model:

— the law of conservation of energy;
— Newton's laws of motion;
— Coulomb's law of friction;
— the principle of minimum potential energy;
— the principle of maximum dissipation;
— Poisson's decomposition into compression and expansion phases;
— Lankarani's decomposition into intervals of sliding and rolling;
— velocity dependence of the COR;
— prohibition of attractive normal impulses;
— direction variation while sliding; and
— coordinate frame invariance.

With these effects considered, PLUS can closely approximate the behaviour of a compliant model; however, computational efficiency cannot be ignored. Therefore, the PLUS model also incorporates the following simplifying assumptions, many of which are shared by other impact models:

— time does not advance;
— bodies neither interpenetrate nor deform;
— the system configuration remains unchanged;
— velocities change discontinuously;
— external forces have no effect;
— dissipation and friction are modelled with empirical coefficients;
— impulses are applied at discrete points on rigid bodies;
— compressions and expansions occur at all points simultaneously; and
— waves propagate instantaneously after each compression or expansion phase.

These modelling simplifications enable rapid calculation of the macroscale consequences of an impact.

### (b) Fundamentals

We consider impacts in spatial multibody systems modelled with generalized configuration *q* and generalized velocity *u*, and containing arbitrary holonomic and non-holonomic constraints. The time, configuration and velocities at the beginning of the impact are known. At the beginning of an impact, the Poisson compression phase is governed by the following velocity-level equations:

2.1*a* $$\begin{array}{rl} M\Delta u-G_{b}^{T}\pi_{b}-G^{T}\pi & =0 \end{array}$$

2.1b $$\begin{array}{rl} G_{b}(u+\Delta u) & =0 \end{array}$$

2.1c $$\begin{array}{rl} \;\text{and}\;G(u+\Delta u) & =\Delta v_{\mathrm{desired}}, \end{array}$$

where $M\in R^{n\times n}$ is the symmetric, positive definite mass matrix; $u\in R^{n}$ is the generalized velocity at the beginning of the impact; $\Delta u\in R^{n}$ is the unknown instantaneous change to *u*; $G_{b}\in R^{m_{b}\times n}$ and $G\in R^{m\times n}$ are the Jacobians of the bilateral and contact constraints; $\pi_{b}\in R^{m_{b}}$ and $\pi \in R^{m}$ are corresponding vectors of unknown constraint-space impulses; and $\Delta v_{\mathrm{desired}}\in R^{m}$ is the desired constraint-space velocity change needed to halt interpenetration. All external forces are assumed to be non-impulsive and, therefore, have no effect on the system dynamics during impact. Note that this formulation accommodates bilateral constraints and ensures that velocity constraints are satisfied at the end of the impact even if *u* did not satisfy the constraints perfectly when the impact began. For clarity, however, and without loss of generality, we assume in the sequel that *Gu*=0 and *m*_*b*_=0. With these assumptions, solving (2.1) for *π* gives the following:

2.2 $$A\pi =\Delta v_{\mathrm{desired}},$$

where $A\in R^{m\times m}≜GM^{-1}G^{T}$ is the constraint-space compliance matrix [28] or Delassus operator [9]. By conceptually magnifying the time axis during impact, we track the accumulation of normal impulses $\pi_{z}\in R$ and tangential impulses $\pi_{xy}\in R^{2}≜{\left[\pi_{x},\pi_{y}\right]}^{T}$ as we proceed through the compression and expansion phases.

### (c) Normal impulses

At the beginning of the compression phase, the impacting bodies will have scarcely begun to interact; in our simple example, the brick vertex will be touching the ground, will have a normal velocity directed towards the ground ($v_{z}^{(\mathrm{start})}<0$), and no impulses will have been applied. The end of the compression phase corresponds to the instant at which the approach velocity has vanished ($v_{z}^{(\mathrm{end})}=0$). Thus, the normal impulse required to proceed to the end of the compression phase can be calculated as follows:

2.3 $$A_{z}\pi =-v_{z}^{\left(\mathrm{start}\right)},$$

where *A*_*z*_=G_*z*_*M*^−1^G^T^ is the row of *A* corresponding to *π*_*z*_. Energy dissipation during impact is caused primarily by wave propagation, and to a lesser extent by localized plastic deformation [3]. Poisson's kinetic restitution hypothesis captures this behaviour in a simple empirical formula relating compression and expansion impulses:

2.4 $$\pi_{z}^{(\exp)}=e\pi_{z}^{\left(\mathrm{comp}\right)},$$

where $\pi_{z}^{(\exp)}$ is the expansion impulse that must be applied, $\pi_{z}^{(\mathrm{comp})}$ is the total normal impulse applied during the compression phase, and 0≤*e*≤1 is Poisson's COR [19].

Newton's kinematic restitution hypothesis relating initial and final velocities is more commonly used than Poisson's, yet is unable to capture important coupled impact effects [7] and is energetically inconsistent in common circumstances [5,6,8]. Stronge's interpretation of the COR as a ratio of energies may be more energetically consistent than Poisson's when employed in simple impact models [7,29]; however, Djerassi [18] concludes that Poisson's hypothesis provides similar results over a broader range of applications. Stronge's approach is also computationally more expensive to implement. For our purposes, Poisson restitution is able to capture the important effects neglected by Newton restitution and, as will be demonstrated in §4, our model reliably exhibits compliant-like behaviour.

**Figure 2.**
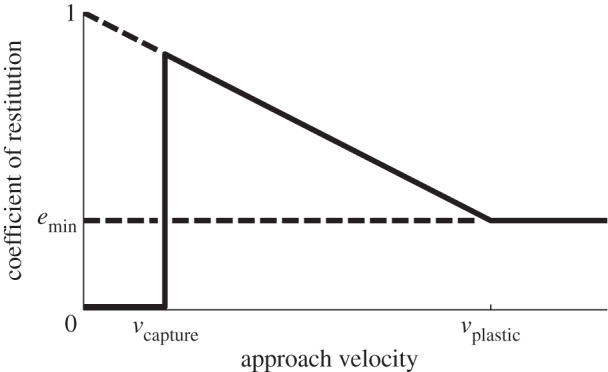
The coefficient of restitution (COR) in our impact model varies with the approach velocity, −*v*_*z*_. The linear relationship and minimum for −*v*_*z*_≥*v*_plastic_ reflect the experimental results reported by Goldsmith [30] and Schiehlen *et al.* [1]. The COR vanishes for −*v*_*z*_<*v*_capture_, as noted by Quinn [32].

It is common practice to treat the COR as being independent of the impact velocity, which is in stark contrast to the linear dependence suggested by empirical observations [1,30]. This linear relationship is modelled as *e*=1−*dv* by Hunt & Crossley [31], where *v* is the impact velocity and *d* is a dissipation coefficient that is a property of the colliding materials. We instead use a velocity-dependent COR that approaches 1 at low impact velocities and reaches a minimum at high velocities where plastic deformation occurs (figure 2), which better approximates experimental results [1,30]. The COR vanishes at very low impact velocities to preserve the behaviour of a compliant model, which will dissipate enough energy to preclude separation at impact velocities below a non-zero threshold [32]—at which point the compliant model predicts continued decreasing oscillations without separation while PLUS predicts steady contact.

Also note that each normal velocity *v*_*z*_ and normal impulse *π*_*z*_ pair must satisfy the following complementarity condition [33]:

2.5 $$0\leq v_{z}⊥\pi_{z}\geq 0,$$

which indicates that either interpenetration is prevented by a normal impulse (i.e. *v*_*z*_=0 and *π*_*z*_>0) or there is neither interpenetration nor application of a normal impulse (i.e. *v*_*z*_>0 and *π*_*z*_=0). We further discuss this complementarity condition in §3.

### (d) Tangential impulses

The compression and expansion phases are each comprised of a sequence of intervals, where an impacting point is either rolling or sliding throughout each interval; the behaviour of a point can change only in the instant between intervals. A point will be rolling if its tangential velocity magnitude at the beginning of the interval is smaller than a sliding-to-rolling transition velocity parameter ($∥v_{xy}^{(\mathrm{start})}∥<v_{\mathrm{transition}}$) and a tangential impulse *π*_*xy*_ can be applied to prevent sliding without violating the friction cone constraint:

2.6 $$∥\pi_{xy}∥\leq \mu \pi_{z},$$

where *μ* is the coefficient of sliding friction. Note that we assume impacts are sufficiently brief to preclude the development of static friction [34]. It is possible that the higher static coefficient would be more realistic for contacts that are already rolling at the beginning of an impact; however, we currently apply the dynamic (sliding) coefficient at all points. The tangential impulse corresponding to rolling points is calculated as follows:

2.7 $$A_{xy}\pi =-v_{xy}^{(\mathrm{start})},$$

which is analogous to (2.3) and drives small residual tangential velocities ($∥v_{xy}^{\left(\mathrm{start}\right)}∥<v_{\mathrm{transition}}$) to zero in a momentum-conserving way, in keeping with Newton's laws of motion.

**Figure 3.**
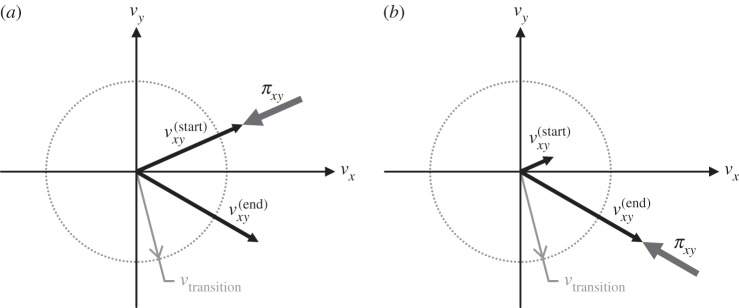
The sliding friction impulse *π*_*xy*_ opposes the tangential velocity *v*_*xy*_ (*a*) at the beginning of the interval if $∥v_{xy}^{(\mathrm{start})}∥\geq v_{\mathrm{transition}}$, and (*b*) at the end of the interval if $∥v_{xy}^{(\mathrm{start})}∥<v_{\mathrm{transition}}$, where *v*_transition_ is the sliding-to-rolling transition velocity parameter.

If a tangential impulse *π*_*xy*_ cannot prevent sliding without violating the friction cone constraint, then the point will slide and we apply a friction impulse that opposes the sliding velocity direction $\hat{s}$:

2.8 $$\pi_{xy}=-\mu \pi_{z}\hat{s},$$

which respects Coulomb's law and the principle of maximum dissipation [3]. When the tangential velocity at the beginning of the interval is of a sufficient magnitude to provide a reliable direction, the sliding friction impulse opposes this velocity; in the cases of *impending slip*, where the tangential velocity at the beginning of the interval is small and may be dominated by numerical noise, the sliding friction impulse opposes the tangential velocity at the *end* of the interval (figure 3):

2.9 $$\hat{s}=\left\{\begin{array}{ll} \frac{v_{xy}^{(\mathrm{start})}}{∥v_{xy}^{\left(\mathrm{start}\right)}∥}, & \mathrm{if}\;∥v_{xy}^{\left(\mathrm{start}\right)}∥\geq v_{\mathrm{transition}}\\ \frac{v_{xy}^{\left(\mathrm{start}\right)}+A_{xy}\pi }{∥v_{xy}^{\left(\mathrm{start}\right)}+A_{xy}\pi ∥}, & \mathrm{otherwise}. \end{array}\right.$$

Because, by construction, the tangential velocity evolves linearly from $v_{xy}^{\left(\mathrm{start}\right)}$ to $v_{xy}^{\left(\mathrm{end}\right)}$ throughout the interval, opposing $v_{xy}^{\left(\mathrm{end}\right)}$ is nearly equivalent to opposing $v_{xy}^{\left(\mathrm{start}\right)}$ in the case of impending slip.

### (e) Calculating incremental impulses

To calculate the impulse that must be applied to proceed to the next interval, we must first assemble and solve a linear system of equations. Returning to our simple example, suppose the brick vertex has just begun colliding with the ground in a direct impact with high friction and no tangential velocity. In this case, we would combine (2.3) and (2.7) to solve for *π*:

2.10 $$A\pi =-v^{(\mathrm{start})},$$

where *π*=[*π*_*x*_,*π*_*y*_,*π*_*z*_]^T^. In an oblique impact, on the other hand, where the impacting point has a non-negligible velocity in the tangent plane at the beginning of the impact, the point would begin sliding rather than rolling:

2.11 $$\left[\begin{array}{ccc} 1 & 0 & \mu {\hat{s}}_{x}\\ 0 & 1 & \mu {\hat{s}}_{y}\\ & A_{z} & \end{array}\right]\left\{\begin{array}{c} \pi_{x}\\ \pi_{y}\\ \pi_{z} \end{array}\right\}=\left\{\begin{array}{c} 0\\ 0\\ -v_{z}^{(\mathrm{start})} \end{array}\right\},$$

where the rolling equations (2.7) have been replaced with the sliding equations (2.8). We refer to linear systems of the forms shown in (2.10) and (2.11) collectively as *Bπ*=*w*. In this simple example, we have no redundant constraints and can readily solve for the impulse *π* that advances the impact process to the end of the compression phase. Note that this linear system is implicit in *π* when one or more points are in impending slip, in which case we solve for *π* using Newton's method supplied with an analytical Jacobian; convergence is very fast in practice.

To capture the rich structure of the impact process, we apply to the system only a fraction 0<*α*≤1 of the calculated impulse *π*. We apply the entire calculated impulse (i.e. *α*=1) and proceed to the end of the current impact phase only if two conditions are met: (i) sliding does not cease, and (ii) the sliding direction does not change substantially. The first condition involves detecting transitions from sliding to rolling, which are found by identifying sliding points whose tangential velocity magnitude approaches zero:

2.12 $$∥v_{xy}^{(\mathrm{start})}+\alpha A_{xy}\pi ∥<v_{\mathrm{transition}}.$$

**Figure 4.**
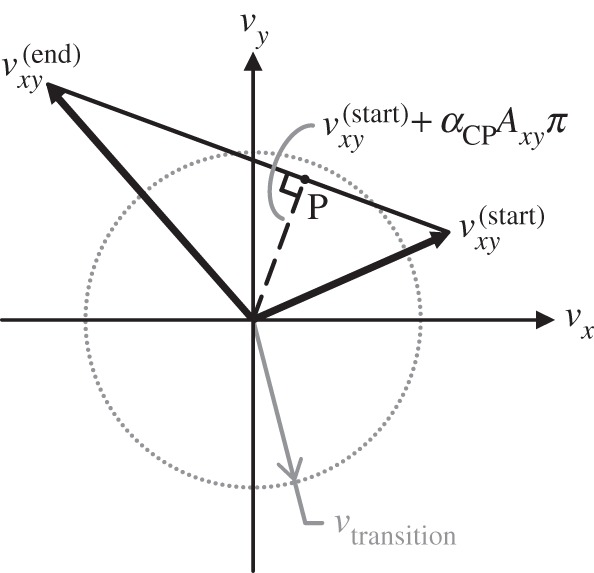
Transitions from sliding to rolling are detected geometrically. The line segment connecting the tangential velocity vector at the beginning of the interval to that obtained upon applying the computed impulse *π* tracks the evolution of the tangential velocity over interval step lengths 0≤*α*≤1. The closest point on this line segment to the origin (P) is reached upon taking a step of length *α*_CP_. A transition to rolling may occur if $∥v_{xy}^{(\mathrm{start})}+\alpha_{\mathrm{CP}}A_{xy}\pi ∥<v_{\mathrm{transition}}$.

In practice, this condition will rarely be observed at the end of the interval; instead, the sliding velocity will have been reversed by the applied tangential impulse, meaning the transition to rolling was missed. We must find *α*<1 corresponding to the sliding-to-rolling transition. For 0≤*α*≤1, $v_{xy}^{\left(\mathrm{start}\right)}+\alpha A_{xy}\pi$ defines a line segment connecting the tangential velocity vector at the beginning of the interval to that obtained upon applying the entire computed impulse *π*. We calculate the interval step length *α*_CP_ required to reach the closest point on this line segment to the origin, which represents the tangential velocity of minimum magnitude over the interval (figure 4):

2.13 $$\alpha_{\mathrm{CP}}=\frac{-v_{xy}^{\left(\mathrm{start}\right)}\cdot (v_{xy}^{\left(\mathrm{end}\right)}-v_{xy}^{\left(\mathrm{start}\right)})}{∥v_{xy}^{\left(\mathrm{end}\right)}-v_{xy}^{\left(\mathrm{start}\right)}{∥}^{2}}=-\frac{v_{xy}^{\left(\mathrm{start}\right)}\cdot A_{xy}\pi }{∥A_{xy}\pi {∥}^{2}}.$$

If $∥v_{xy}^{(\mathrm{start})}+\alpha_{\mathrm{CP}}A_{xy}\pi ∥<v_{\mathrm{transition}}$, then we set *α*=*α*_CP_ and proceed to the next interval; otherwise, we check the second condition on the interval step length, described below. In practice, we set *α*=1 if $∥v_{xy}^{\left(\mathrm{end}\right)}∥<v_{\mathrm{transition}}$, regardless of the value of *α*_CP_, to avoid computing a small terminal interval. Note that rolling-to-sliding transitions are detected when determining the active set, a discussion of which we defer to §3.

The second condition on the interval step length is dictated by the angle through which the tangential velocity rotates. Because the friction impulse applied during an interval of sliding opposes the tangential velocity at the *beginning* of the interval, we must update $\hat{s}$ in (2.8) if the tangential velocity undergoes a substantial change in direction. We permit a predetermined change *θ*_max_ in the direction of each sliding velocity, and truncate the interval to avoid exceeding this limit at any sliding point. Many existing approaches assume sliding directions remain constant throughout impact [11,35]; as will be shown in §4*a*, this assumption is poor even in ostensibly simple scenarios. Impact forces can be very large, so applying an impulse in the wrong direction can incur serious macroscale errors, significantly affecting the subsequent simulation.

**Figure 5.**
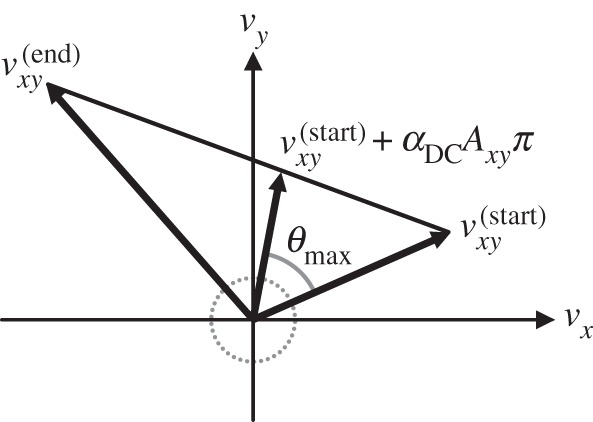
The interval step length required to reach the maximum permissible sliding direction change is computed geometrically. The line segment connecting the tangential velocity vector at the beginning of the interval to that obtained upon applying the computed impulse *π* tracks the evolution of the tangential velocity over interval step lengths 0≤*α*≤1. The maximum permissible sliding direction change *θ*_max_ is reached upon taking a step of length *α*_DC_. The interval step length may be limited by the direction change constraint if 0<*α*_DC_<1.

The interval step length *α*_DC_ corresponding to the maximum permissible sliding direction change is computed geometrically (figure 5):

2.14 $$\cos(\theta_{\max})=\frac{v_{xy}^{\left(\mathrm{start}\right)}\cdot (v_{xy}^{\left(\mathrm{start}\right)}+\alpha_{\mathrm{DC}}A_{xy}\pi )}{∥v_{xy}^{\left(\mathrm{start}\right)}∥\;∥v_{xy}^{\left(\mathrm{start}\right)}+\alpha_{\mathrm{DC}}A_{xy}\pi ∥},$$

which is a quadratic equation in *α*_DC_ yielding two solutions, one positive and one negative. We retain the positive solution bound to the interval [*α*_min_,1], where *α*_min_ is a (small) minimum permissible step length. Although the sliding friction impulse exactly opposes the tangential velocity at only the beginning of the interval, this error can be reduced arbitrarily by reducing *θ*_max_. As will be shown in §4, smaller values of *θ*_max_ produce behaviour that converges to that of a compliant contact model; larger values of *θ*_max_ produce increasingly coarse approximations thereof. In practice, many impacts require only a single interval, and for those in which the direction changes rapidly, considerable improvement in the final impulse direction is achieved with a small number of intervals. Also, unlike the linear complementarity formulations that employ polyhedral approximations of the friction cone [4,11,28], whose complexity increases exponentially as the anisotropism of the friction behaviour is reduced [36], reducing *θ*_max_ increases the number of intervals that will be computed without increasing the complexity of computing each interval.

Interval step length *α* is analogous to a variable integrator step size, where sliding-to-rolling events are isolated by *α*_CP_ and the error estimate of *α*_DC_ prevents large deviations between sliding directions and the directions in which friction impulses are applied. As shown in (2.13) and (2.14), *α*_CP_ and *α*_DC_ are easily computed, and provide a low-cost method of determining whether additional intervals are necessary. As might be expected, the interval step length applied when multiple points are impacting is simply the minimum of the step lengths calculated for each point. We elaborate on the handling of multi-point impacts in §3.

## 3. The PLUS model. Part II: simultaneous impacts

Here, we describe the components of the PLUS model pertaining to the simulation of simultaneous impacts. We first discuss the handling of redundant constraints and define the constrained optimization problem being solved for each interval, which is a generalization of the equations presented in §2. We then describe our strategies for determining the state of each impacting point during each interval and for processing new impacts induced by the application of impulses during the collision. The algorithm has been implemented in the Simbody multibody dynamics library [17]; a summary of the algorithm can be found in the appendix (see the electronic supplementary material). Again, for clarity and without loss of generality, we focus our current discussion on collisions between the vertices of a brick and a horizontal ground plane.

### (a) Simultaneity and redundancy

**Figure 6.**
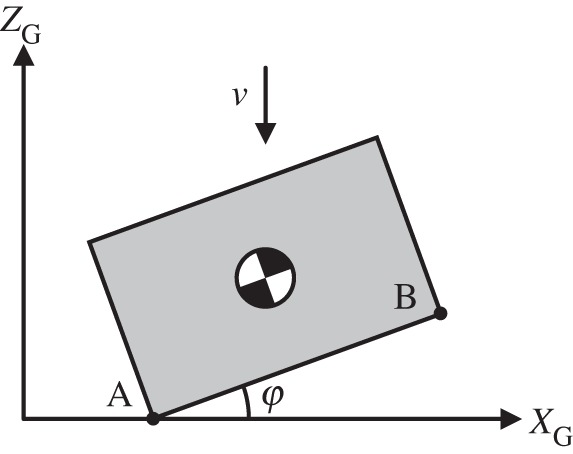
Impact is triggered by strict interpenetration, but any vertex below or within a specified distance of the ground may be participating in the ensuing impact event. In the situation depicted here, we would expect the block to rebound directly upward if *φ*=0 and, indeed, if |*φ*|<*ϵ* for some small tolerance *ϵ*>0. Considering this proximity tolerance is necessary to avoid sensitivity to numerical noise, to maintain symmetry and to preserve the load-spreading behaviour of a compliant contact model—in this case, spreading the load between vertices A and B.

An impact event is triggered when two previously separated, unilaterally constrained geometric features are first seen to overlap. Although a particular pair of features initiates the event, it is possible for two or more feature pairs to collide simultaneously. Treating these collisions as consecutive pairwise impacts would produce a solution that is dependent on the arbitrary order in which the impacts were processed [37]. Consequently, we allow any feature pair closer than a specified tolerance to participate in the ensuing impact, as depicted in figure 6. Our assumption is that the given tolerance represents the known uncertainty with which positions are determined during the simulation. If consideration of this uncertainty indicates that a feature pair may be touching, we label that pair *proximal*; all proximal pairs are considered simultaneously in the impact calculation. Without this notion of a proximity tolerance, the simulation would be sensitive to numerical noise, would lose symmetry unexpectedly, and would no longer produce behaviour resembling that of a compliant contact model under the same circumstances. In the case of the brick–ground collision considered here, all vertices below or within a specified distance of the ground are labelled proximal, and we will use the shorthand ‘proximal point’ or ‘contact point’ to refer to the feature pair consisting of a vertex and the ground plane. Not all proximal points will have impulses applied, as will be discussed below.

Proximal points often produce redundant constraints. Consider, for example, a brick colliding squarely with the ground plane. Considering only out-of-plane motion, the brick has just three degrees of freedom (DOFs): one translation and two rotations. Normal constraints at three vertices would be sufficient to fully constrain this motion and elicit the expected rebound, but four vertices would be proximal. Considering all motion of the brick, each proximal vertex could provide three constraints for a total of 12 available to constrain the brick's six DOFs. In the presence of redundant constraints, equations of the form (2.10) and (2.11) are insufficient to specify a unique solution for the impulse vector *π*. Note that this redundancy is an artefact of the rigid-surface assumption; a real brick, or a compliant model of one, has no such redundancy and will spread the load by deforming across the available contact surface. The *principle of minimum potential energy* [38], a fundamental aspect of the theory of elasticity, states that such deformations will be distributed to minimize the overall stored potential energy, or strain energy. For linearly elastic materials, energy storage is quadratic in displacement. Thus, if we choose a finite set of contact points with which to represent the contacting surfaces and place identical linear springs at each contact, we will find that displacements are distributed in a least-squares manner at equilibrium because the potential energy of displacement would then be minimized.

This observation forms the basis for our choice of a unique solution in the presence of redundant constraints: we find the least-squares solution for *π* in each interval. While computing the true solution requires a detailed compliant contact model, we argue that the least-squares solution is a much better choice for a rigid approximation than is the commonly used numerical selection of an inconstant, non-redundant subset of the available constraints [2]. The least-squares solution exhibits two qualitative features of compliant contact that are absent in the alternative approach: (i) load spreading to minimize potential energy and (ii) solution stability from one step to the next. In contrast, numerical pivoting can place a different, seemingly arbitrary, subset of the constraints under load at each step, whereas other constraints show zero load. Furthermore, the least-squares solution for *π* can be obtained efficiently using a complete orthogonal factorization [39]. Our approach is similar to the Moore–Penrose pseudo-inverse method discussed by Wojtyra [40] for joint redundancies. Mirtich [41] suggested resolving contact redundancies similarly, but did not implement this approach in his work. To the best of our knowledge, use of a least-squares solution has not been reported previously for impact methods, nor has its connection to the principle of minimum potential energy been noted. While no rigid method can be said to yield the ‘true’ answer, use of a load-spreading solution can at least achieve a solution that is consistent with the principles of contact mechanics, while remaining within the permitted computational budget.

In the general case, we solve the least-squares problem given in table 1 to compute *π* in each interval. We assume that the local impact processes occurring at all participating points evolve in synchrony [42]. To assemble the least-squares problem, we must first classify each proximal point as either compressing, expanding or *observing* (i.e. not yet participating in the impact), and determine whether each non-observing point is rolling, sliding or in impending slip. This problem is the focus of the next section.

**Table 1.** The minimization problem being solved in each interval of the PLUS algorithm. Minimizing the 2-norm of impulse vector *π* in the presence of redundant constraints preserves the load-spreading behaviour and solution stability of a compliant contact model. Each proximal point is always either compressing, expanding or observing; non-observing points are always either rolling, sliding or in impending slip in the tangent plane. *A*_*z*[*k*]_ and *A*_*xy*[*k*]_ are the rows of *A* corresponding to the normal and tangential impulses applied to the *k*th proximal point. All velocities *v* and the remaining expansion impulses $\pi_{z[k]}^{(\exp)}$ are updated after each interval.

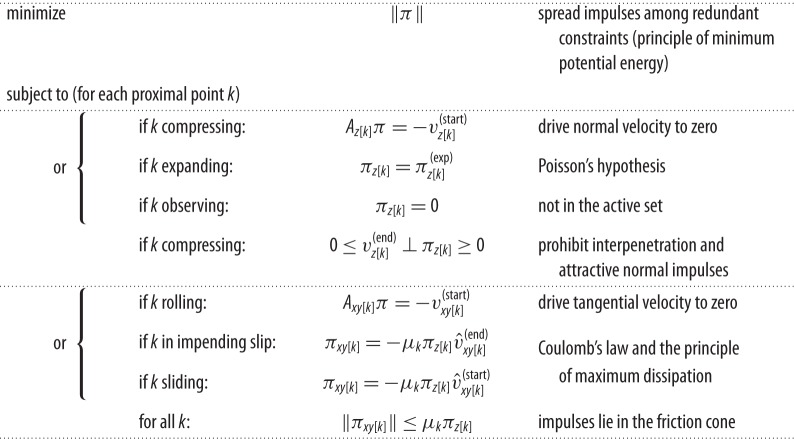

### (b) Propagation and active set selection

In §2, we modelled an impact as a compression phase followed by an expansion phase. When two or more points are proximal, however, new impacts can be induced by the application of impulses during the collision. These dispersion or scattering effects model the propagation of waves throughout the system [43–45], which must be considered to obtain unique, energetically consistent results [46]. Note that proximal points are so labelled based solely on their position: because velocities change throughout the impact, a proximal point that is originally retreating may later be approaching. In particular, note that there is no constraint preventing a point from acquiring a negative normal velocity (i.e. impacting) while expanding or observing. Thus, we adopt the notion of impact *rounds* and label each proximal point as either compressing, expanding or observing for the duration of each round. Determining which points are observing is straightforward: they have non-negative normal velocities. Expanding points are those that were compressing in the previous round, had a non-zero impulse applied in that round, and have a non-zero COR. All the remaining points are classified as compressing; however, not all compressing points will generate impulses, since they must each satisfy the complementarity condition (2.5). If satisfaction of (2.5) results in application of an impulse, then the compressing point is said to be *active*. Determining the active compressing points is further complicated by the detection of rolling-to-sliding transitions within each round, and by the unknown sliding directions of points in impending slip.

We use an active set strategy [47] to determine the state of each normal constraint for compressing points and each rolling constraint for non-observing points, as described in algorithm 1 (see appendix in the electronic supplementary material). For small problems, an exhaustive search can be used to determine the optimal active set. This approach is impractical in general, but useful for validating more efficient methods. The heuristic we will present is not guaranteed to converge to the optimum; however, our experience is that it quickly produces optimal or near-optimal solutions in practice. Recall that we wish to obtain the global optimum for the least-squares problem given in table 1. Our strategy seeks the solution that contains the largest number of active constraints, since the least-squares solution will be the one with the most non-zero entries in *π*, spreading the load across the redundant constraints. Thus, our active set strategy is designed to bias the solution towards preserving as many constraints as possible. Note that bilateral constraints are easily incorporated into this scheme because they are always active.

Our active set selection algorithm begins by identifying those points whose state is known. The classifications of compressing, expanding and observing points are made at the beginning of each round as indicated above. Further, at the beginning of each interval, any non-observing point whose current tangential velocity is large (i.e. $∥v_{xy}^{\left(\mathrm{start}\right)}∥\geq v_{\mathrm{transition}}$) is sliding. (This condition reflects the fact that every oblique impact begins with a sliding interval because, in reality, normal forces—and, therefore, frictional forces—take some time to develop.) All remaining constraints are now assumed to be active—that is, normal non-penetration constraints are enforced for compressing points, and tangential rolling constraints are enforced for all points that are not sliding. We assemble a linear system analogous to (2.10) and (2.11), and obtain a least-squares solution for *π*. The solution is then examined for constraint violations: the magnitude of the tangential impulse applied to each rolling point must lie within its friction cone (i.e. ∥*π*_*xy*[*k*]_∥≤*μ*_*k*_*π*_*z*[*k*]_ for all rolling points *k*), and the normal impulse applied to each point in compression must be repulsive (i.e. *π*_*z*[*k*]_≥0 for all compressing points *k*). The constraint associated with the largest violation is identified, as is the point *p*_ℓ_ associated therewith. If *p*_ℓ_ was assumed to be rolling, we conclude that the coefficient of friction is insufficient to prevent the point from sliding, so *p*_ℓ_ is now considered to be in impending slip and its rolling constraints are removed from the active set. If *p*_ℓ_ was assumed to be sliding or in impending slip while compressing, we conclude that no normal impulse is necessary to satisfy complementarity condition (2.5), and the normal constraint associated with *p*_ℓ_ is removed from the active set. This process is repeated until all active constraints satisfy the friction cone (2.6) and normal impulse sign conditions, and is guaranteed to terminate because the size of the active set is strictly decreasing with each iteration. The heuristic can fail to satisfy some velocity conditions, however, in which case the violating point is treated as a new impact in the next round. The outer loop of the PLUS impact event handler is summarized in algorithm 2 (see appendix in the electronic supplementary material), an implementation of which is available in the Simbody multibody dynamics library.

## 4. Simulation results

Here, we demonstrate the features of the PLUS impact model using four examples. We first examine a collision between a brick and the ground plane where contact occurs at a single point. This first example is used to illustrate the rich behaviour that can occur even in ostensibly simple scenarios, and to compare the behaviour of our model with that of a compliant contact model. In the second and third examples, we explore simultaneous impacts where all points evolve in unison and where new impacts are induced during a collision. In the final example, we discuss computational complexity and provide timing comparisons using our implementation in Simbody.

Simbody's compliant contact model [17] is based on Hertzian elastic deformation theory [48], the dissipation model of Hunt & Crossley [31] and a Stribeck friction model [49]. Hertzian contact theory for a sphere relates displacement *z* and normal force *f*_Hz_ as follows:

4.1 $$f_{\mathrm{Hz}}=\frac{4}{3}\sqrt{R}Ez^{3/2},$$

where *R* is the relative curvature of the colliding bodies and *E* is the effective plane strain modulus. The model of Hunt and Crossley adds a dissipation term to (4.1):

4.2 $$f_{\mathrm{HC}}=f_{\mathrm{Hz}}(1+\frac{3}{2}c\dot{z}),$$

where *c* is the effective dissipation coefficient. The friction force is modelled as a three-segment splined function of the relative tangential velocity, which is parametrized by static, dynamic and viscous coefficients of friction, and a transition speed at which static friction reaches its peak value (see fig. 5 in Sherman *et al.* [17]). In the following examples, we use equal static and dynamic coefficients of friction, no viscous friction, and a transition speed of 0.01 m s^−1^ in the compliant contact model.

### (a) Example 1: impact at a single point

**Figure 7.**
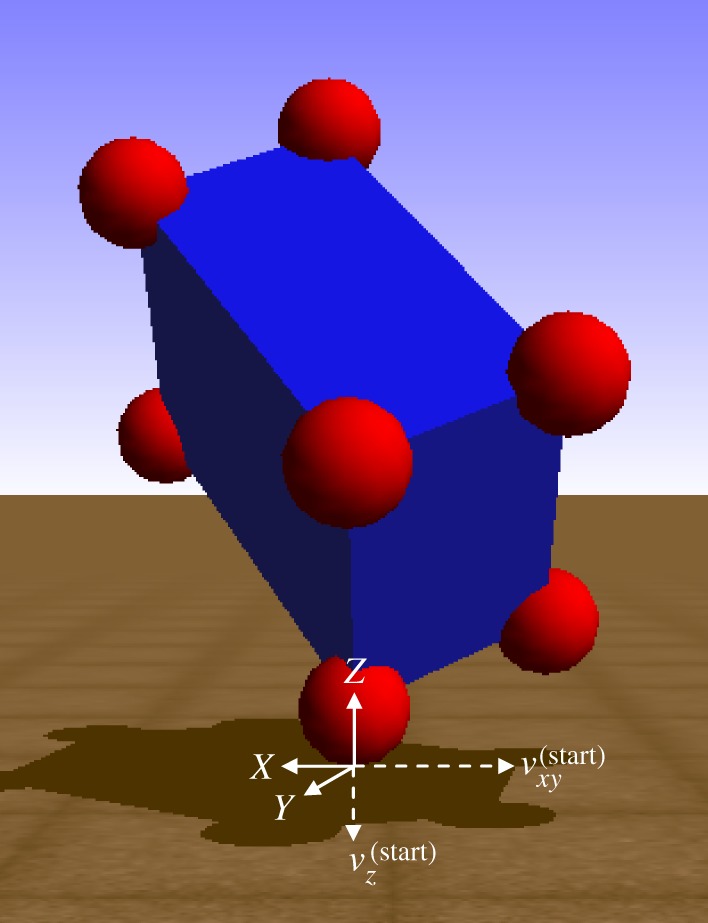
A single-point collision between a childproof brick and the ground plane in Simbody. The point slides at the beginning of the impact process owing to its non-negligible relative tangential velocity. Depending on the coefficient of friction, the point may continue sliding throughout the impact, or it may transition to rolling in either the compression phase or the expansion phase.

We first consider the scenario depicted in figure 7, where a single point on a childproof brick collides with the ground plane. Spheres have been welded to the vertices of the brick to facilitate direct comparison with Simbody's compliant contact model, since point contacts are regions of infinite stress in such models. The orientation of the brick, shown in figure 7, is obtained through body-fixed rotations of *π*/4 rad about the *x*-axis and *π*/6 rad about the *y*-axis; the remaining parameters are given in electronic supplementary material, table S1 (see appendix in the electronic supplementary material). To facilitate comparison, the COR in the PLUS impact model is chosen to match the effect of the dissipation coefficient set in the compliant contact model. All simulations with the compliant model are performed using the error-controlled Runge–Kutta–Merson integrator [50] with an accuracy of 10^−5^.

**Figure 8.**
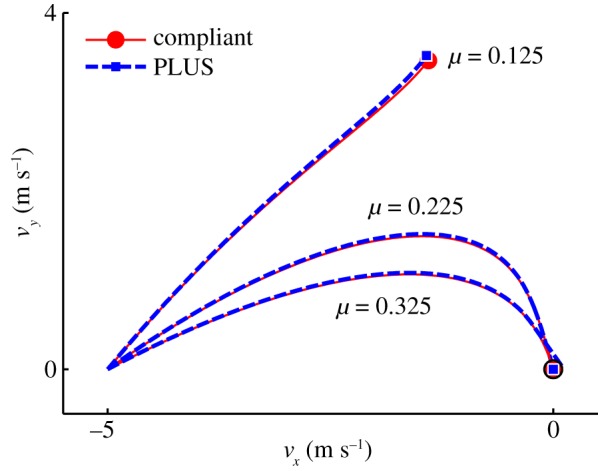
Trajectories of tangential velocities during the single-point impact scenario of example 1, shown in figure 7, using a compliant contact model (solid) and the PLUS impact model (dashed). The three coefficients of friction correspond to cases in which the point slides throughout the collision (*μ*=0.125), transitions to rolling in the expansion phase (*μ*=0.225) and transitions to rolling in the compression phase (*μ*=0.325). The black circle at the origin is of radius *v*_transition_. When *μ*=0.325, note that the sliding-to-rolling transition occurs when the trajectory of the tangential velocity is tangent to this circle (not when the trajectory passes through the origin) to ensure robustness to numerical noise and to reduce the number of short terminal sliding intervals.

**Figure 9.**
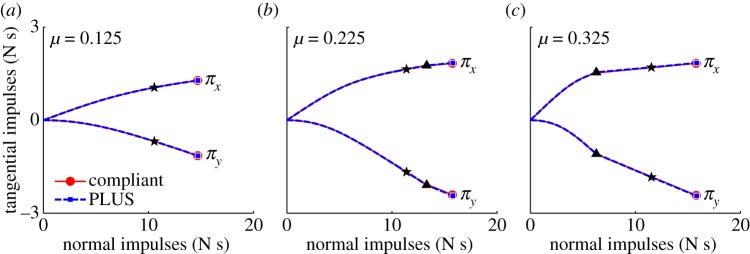
Impulses accumulated during the single-point impact scenario of example 1, shown in figure 7, using a compliant contact model (solid) and the PLUS impact model (dashed). Circles and squares indicate the total impulses accumulated over each collision, stars indicate the points at which compression ends and expansion begins, and triangles indicate transitions from sliding to rolling. (*a*) *μ*=0.125 and the point slides throughout the collision; (*b*) *μ*=0.225 and the point transitions to rolling in the expansion phase and (*c*) *μ*=0.325 and the point transitions to rolling in the compression phase.

**Figure 10.**
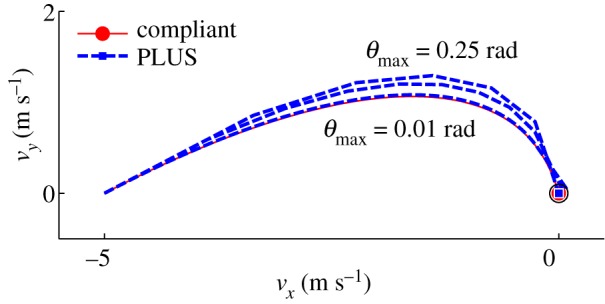
Trajectories of tangential velocities during the single-point impact scenario of example 1, shown in figure 7, using a compliant contact model (solid) and the PLUS impact model (dashed). A close match is obtained for *θ*_max_=0.01 rad, but 116 intervals are required. Moderately less accurate solutions are obtained for *θ*_max_=0.15 rad (11 intervals) and *θ*_max_= 0.25 rad (eight intervals).

As shown in figure 8, the tangential velocities computed using the PLUS model evolve over nearly identical trajectories as those computed using our compliant contact model for all three collisions. The impulses accumulated by the PLUS model during each collision, shown in figure 9, are also very close approximations of those observed when using the compliant contact model. Note the substantial change in the tangential velocity direction over the duration of each collision; the fixed-direction, single-interval strategy commonly employed is clearly a poor approximation of this behaviour, except for the *μ*=0.125 case in which sliding persists for the entire impact. Also note that the accumulated impulses evolve over clearly nonlinear trajectories, which is in contrast to the linear process diagrams used in earlier work to model impacts in planar systems [6,20,24,25]. The small deviations in the accumulated impulses and tangential velocities can be attributed to the infinite stiffness assumption made by the PLUS model, which ignores the small configuration changes that occur when the compliant model is used. In practice, we set the maximum sliding direction change permitted in a single interval to a value larger than 0.01 rad; as shown in figure 10, the performance of the PLUS model degrades predictably and non-catastrophically as *θ*_max_ increases, with qualitatively correct behaviour obtained even with very few intervals. Note that the impulses that oppose these changing directions are accumulated to produce a single total impulse; the direction of this cumulative impulse has a large effect on the subsequent behaviour of the simulation. PLUS chooses the number of intervals adaptively so there is no additional cost if the sliding velocity does not change direction, but when there are significant changes, macroscopically correct behaviour is obtained at a very modest cost.

### (b) Example 2: simultaneous impacts in unison

**Figure 11.**
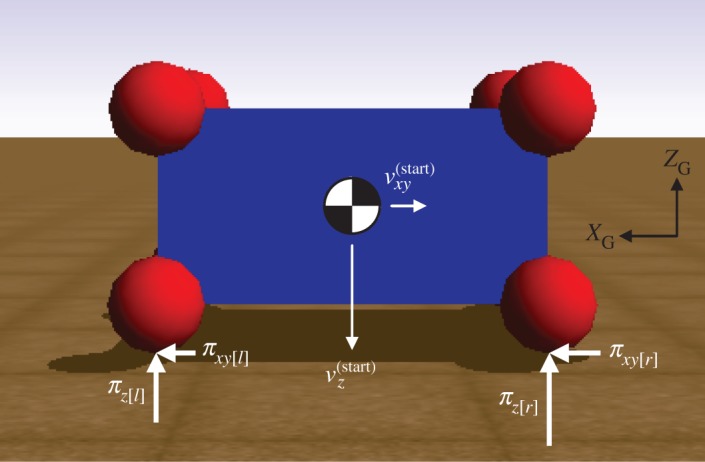
A multi-point collision between a childproof brick and the ground plane in Simbody. All four points are initially sliding and transition to rolling in the common expansion phase. Note that consideration of numerical tolerances is necessary to ensure all four points participate in the impact simultaneously and to preserve symmetry in this scenario.

In the previous example, a single point underwent an impact involving a compression phase followed by an expansion phase. We now consider the scenario depicted in figure 11, where four points on a childproof brick collide with the ground plane simultaneously. As discussed in §3, points that collide simultaneously evolve through their respective impact processes simultaneously. Because the sliding direction does not change in this example, the value of the maximum sliding direction change parameter (*θ*_max_) is immaterial. We use a stiffness of 10 GPa and a dissipation coefficient of 0.15 in the compliant contact model. The minimum COR in our PLUS model is adjusted to approximate the post-collision normal velocities obtained using the compliant model; we set *e*_min_=0.414. The coefficient of friction is *μ*=0.2, and the pre-impact velocity of all four points is *v*^(start)^=[−2,0,−8.84]^T^; all other parameters are given in electronic supplementary material, table S1 (see appendix in the electronic supplementary material).

**Figure 12.**
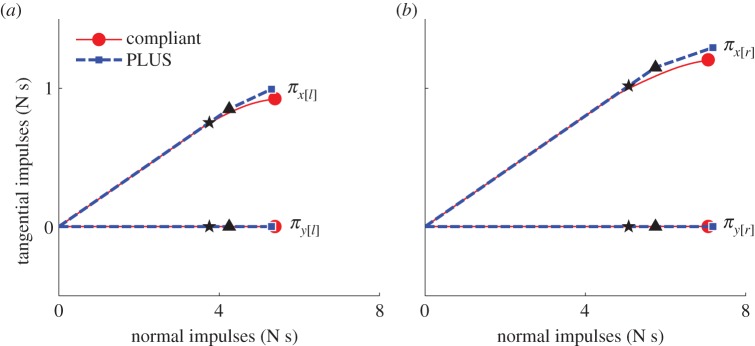
Impulses accumulated during the multi-point collision scenario of example 2, shown in figure 11, using a compliant contact model (solid) and the PLUS impact model (dashed). Circles and squares indicate the total impulses accumulated over the collision, stars indicate the points at which compression ends and expansion begins, and triangles indicate transitions from sliding to rolling. Impulses applied to points shown on the (*a*) left and (*b*) right of figure 11.

**Figure 13.**
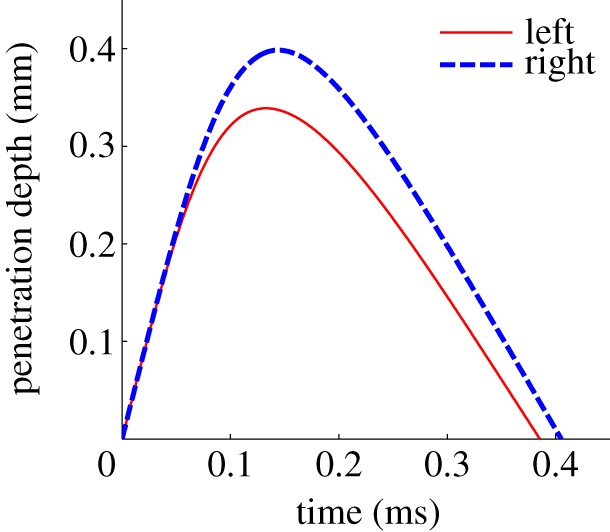
Penetration depth of the points shown on the left (solid) and right (dashed) of figure 11 over the duration of the collision, simulated using a compliant contact model. The moment owing to friction forces causes the brick to rotate clockwise during the impact, driving the points on the right side further into the ground, thereby increasing both the normal and tangential forces applied to these points in comparison with the points on the left side of the brick.

As shown in figure 12, the impulses accumulated by the PLUS model during the collision closely approximate those accumulated by the compliant contact model. The differences between the impulses applied to the left and right points when using the compliant model are due to the differing amounts of interpenetration occurring at these points, as shown in figure 13. Recall that the PLUS model assumes the configuration remains fixed during a collision and computes the impulses that would be applied if the materials were infinitely stiff, hence the small discrepancy in figure 12.

### (c) Example 3: simultaneous and induced impacts

**Figure 14.**
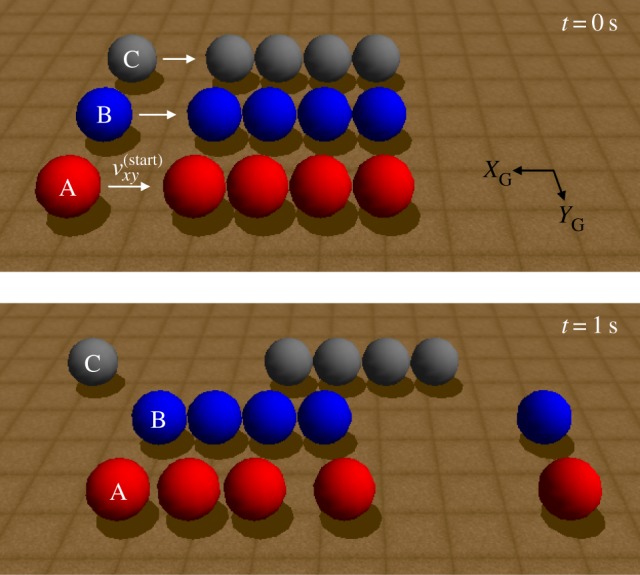
A frictionless ball collides with a row of four identical, touching, stationary balls in Simbody. Three strategies for simulating this scenario are shown: using a compliant contact model (row ‘A’), using our PLUS model (row ‘B’), and using an impact model in which all proximal points participate in the impact event, regardless of their relative velocities (row ‘C’). Only the PLUS model approximates the behaviour of the compliant model, demonstrating the benefit of modelling propagation delays using impact rounds.

We next consider the scenario illustrated in figure 14, where we compare the behaviour of a compliant contact model, our PLUS impact model, and an impact model that assumes each impact event consists of a single compression phase followed by a single expansion phase. All balls are frictionless with radius 0.25 m and mass 1 kg. We use a stiffness of 10 GPa in the compliant case with no dissipation; the COR in the other two cases is 1. The pre-impact velocity is *v*^(start)^=[−2,0,0] m s^−1^. As shown in figure 14, the PLUS model (row ‘B’) approximates the behaviour of the compliant model (row ‘A’), and demonstrates the benefit of modelling propagation delays using impact rounds. If no propagation delays are considered (row ‘C’), the four stationary balls behave as a single ball of equivalent mass and produce a solution that is qualitatively undesirable [11], consisting of a single compression phase followed by a single expansion phase. The sequence of impact rounds generated by the PLUS model is shown in table 2. Note that *all* impacts are not treated sequentially: if several points have negative relative velocities, compression and expansion impulses may be applied to all points simultaneously, as seen in the previous example.

**Table 2.** Impact rounds generated by the PLUS model for the scenario of example 3, shown in figure 14. Proximal points with non-negative normal velocities are observers and have no impulses applied in the current round, but may participate in later rounds as their velocities evolve. In this scenario, the impact process consists of five rounds as the initial impulse propagates through the row of balls, inducing new impacts.

balls	round 1	round 2	round 3	round 4	round 5
1 and 2	compressing	expanding	observing	observing	observing
2 and 3	observing	compressing	expanding	observing	observing
3 and 4	observing	observing	compressing	expanding	observing
4 and 5	observing	observing	observing	compressing	expanding

### (d) Example 4: computational efficiency

**Figure 15.**
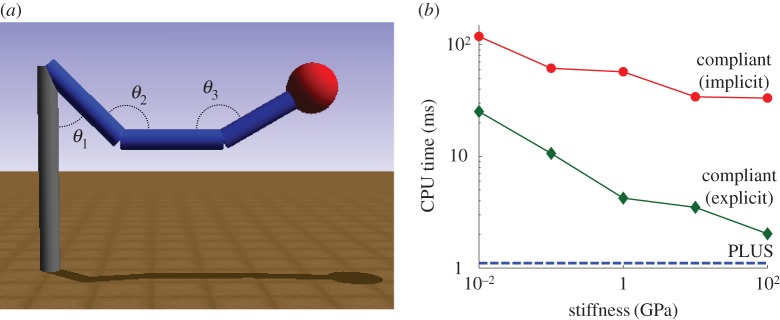
Impact between a triple pendulum and the ground plane: (*a*) initial configuration, and (*b*) CPU time as material stiffness varies (log scales). The PLUS model performs well in comparison with the compliant model. The CPU time required by the compliant model decreases as stiffness increases because less interpenetration occurs and, therefore, less simulation time elapses during stiffer collisions. All simulations were performed on one core of a 3.4-GHz Intel Core i7-3770 processor.

In the final example, we compare the computational efficiency of the PLUS impact model with our compliant contact model, just for the impact portion of a simulation. The time spent in persistent contact, which can be large, is not included here because PLUS treats only impacts. In this example, we simulate a triple pendulum as a sphere attached to its terminus impacts the ground plane. The pendulum is suspended 2 m above the ground and consists of three pin-connected rigid cylinders of length 1 m, radius 0.1 m and mass 1 kg. A sphere of radius 0.25 m, mass 1 kg and friction *μ*=0.1 is welded to the free end of the third cylinder. We use a COR of 1 in the PLUS model and no dissipation in the compliant model. The stiffness of the sphere is varied from 0.01 to 100 GPa in the compliant model. The pendulum falls under the force of gravity from the configuration shown in figure 15*a*, where *θ*_1_=*π*/4 , *θ*_2_=3*π*/4 and *θ*_3_=5*π*/6 rad; the sphere slides throughout the collision. We record the CPU time required to advance through the impact using the PLUS model (in which time does not advance), and using the compliant model with time advanced using variable-step implicit and semi-explicit first-order Euler integrators. For each compliant simulation, the integration accuracy (and, thus, the number of steps) is adjusted to obtain less than 1% error in the final height of the sphere. As shown in figure 15*b*, the PLUS model performs well in comparison with the compliant model. Note that, unlike rigid contact, impact is not a stiff problem because the correct solution contains high-frequency content. Any integrator must take small steps to track the rapidly changing solution during a compliant collision; the step size is driven by accuracy requirements rather than stability. Thus, an implicit integrator is a particularly poor choice for simulating collisions because its nonlinear system is expensive to evaluate and converges poorly when the Jacobian changes rapidly during a step, as is the case here. By simulating collisions as instantaneous events, we avoid the expense of integrating through potentially time-consuming interactions while enabling the use of constraints for modelling persisting contact between bodies. PLUS shares this property with other impact models, and does so while producing results that compare favourably with those of a detailed compliant model.

## 5. Conclusion and future work

We presented a new impact model, called PLUS, that approximates the behaviour of a compliant contact model, adheres to a broad set of physical laws and principles of contact mechanics, considers numerical tolerances explicitly, and does so with modest computational expense. Our formulation accommodates simultaneous impacts in spatial multibody systems modelled with generalized coordinates and containing arbitrary holonomic and non-holonomic constraints. Impacts are modelled as sequences of compression and expansion phases that evolve at multiple points simultaneously to distribute loads and preserve symmetry, and induce further impacts to capture wave propagation effects. Each impact phase comprises sliding and rolling intervals that adjust in number as demanded by the scenario. The length of each interval is computed to detect transitions from sliding to rolling, and to ensure the friction impulse opposes the sliding velocity direction to within a specified tolerance. We use a least-squares solution to maximally distribute impulses among redundant constraints, and respect the physical laws, mechanical principles and empirical observations listed in §2a. PLUS produces impulses similar to those of a compliant contact model, but retains the performance advantage of modelling collisions as instantaneous impact events.

We discussed several important practical issues. PLUS is designed to be executed on a computer at high speed, with user-selectable accuracy and with limited numerical precision. These algorithmic considerations have a substantial effect on any numerical model, but are often considered implementation details and are rarely discussed in the literature. We considered the following issues explicitly:

— all computations are performed approximately;
— impacts cannot be isolated perfectly in either time or space;
— small deviations in time or space are not significant;
— position and velocity constraints cannot be satisfied perfectly;
— decaying rebound velocities will never reach zero exactly; and
— sliding-to-rolling transitions must occur at non-zero velocity.

These issues must be addressed by any computational impact model, and we believe they ought to be acknowledged.

While the benefits of our current formulation are clear, so are its limitations. In particular, we note that the active set algorithm we employ may be time-consuming when many points impact simultaneously. Further, despite its success in practice, there is no guarantee that our active set strategy will converge to the optimal solution. We are currently exploring alternate strategies for solving the constrained optimization problem described in table 1, including nonlinear complementarity [26,51] and prox [52] formulations. Also, although modelling wave propagation delays produces results that align well with those of a compliant contact model, as shown in figure 14, induced impacts also incur additional computational cost—which can be substantial if many impact rounds are necessary. In practice, it may be necessary to limit the number of rounds permitted in a single impulse calculation; research is required to determine how to compute the best possible impulse within a limited computational budget. A similar issue is encountered in existing algorithms that employ iterative methods [2,11], but despite having no proof of convergence, these methods have been shown to be effective in practice. Our detailed treatment of impact events is suitable for problems in which impacts are relatively infrequent, but not for problems such as granular media where impacts are ubiquitous, and for which statistical cancellations permit approximate treatment of individual collisions. Finally, while load-spreading in PLUS achieves qualitatively plausible results, it still cannot be claimed to approximate compliant behaviour in general. Further research may yield methods that produce superior numerical results.

Despite these limitations, PLUS represents a significant advance in the simulation of impacts in rigid-body systems, offering a unique combination of fidelity and performance. We have shown that the performance advantage of impact models can be obtained without sacrificing important qualitative and quantitative aspects of time-resolved compliant models. A fast yet macroscopically accurate impact model is essential for many biomechanical and robotic applications, particularly those involving real-time interactivity and optimization. In addition to being computationally demanding, optimization algorithms will exploit any modelling errors that lead to an improved objective, regardless of whether the results are realistic. Thus, for tasks such as optimizing gait controllers and generating human-like behaviours, it is essential to use an impact model that produces realistic impulses in a wide range of situations. We believe that PLUS suits that role well, and should be considered before using a lower-fidelity impact model in scientific and engineering applications. An open-source implementation of PLUS in Simbody is available on GitHub.

## Supplementary Material

Appendix

## Data accessibility

The algorithms developed in this work were implemented in Simbody, an open-source C++ multibody dynamics library available on GitHub at https://github.com/simbody/simbody.

## References

[1] Schiehlen W Seifried R Eberhard P 2006 Elastoplastic phenomena in multibody impact dynamics. Comput. Methods Appl. Mech. Eng. 195, 6874–6890. (doi:10.1016/j.cma.2005.08.011)

[2] Baraff D 1994 Fast contact force computation for nonpenetrating rigid bodies. In Proc. the 21st Annual Conf. on Computer Graphics and Interactive Techniques (SIGGRAPH), Orlando, FL, 24–29 July, pp. 23–34.

[3] Stewart DE 2000 Rigid-body dynamics with friction and impact. SIAM Rev. 42, 3–39. (doi:10.1137/S0036144599360110)

[4] Bhalerao KD Anderson KS Trinkle JC 2009 A recursive hybrid time-stepping scheme for intermittent contact in multi-rigid-body dynamics. ASME J. Comput. Nonlinear Dyn. 4, 041010 (doi:10.1115/1.3192132)

[5] Kane TR Levinson DA 1985 Dynamics: theory and applications. New York, NY: McGraw-Hill.

[6] Wang Y Mason MT 1992 Two-dimensional rigid-body collisions with friction. ASME J. Appl. Mech. 59, 635–642. (doi:10.1115/1.2893771)

[7] Hurmuzlu Y Marghitu DB 1994 Rigid body collisions of planar kinematic chains with multiple contact points. Int. J. Robot. Res. 13, 82–92. (doi:10.1177/027836499401300106)

[8] Djerassi S 2009 Collision with friction; part A: Newton's hypothesis. Multibody Syst. Dyn. 21, 37–54. (doi:10.1007/s11044-008-9126-2)

[9] Glocker C 2013 Energetic consistency conditions for standard impacts. Part I: Newton-type inequality impact laws and Kane's example. Multibody Syst. Dyn. 29, 77–117. (doi:10.1007/s11044-012-9316-9)

[10] Drumwright E Shell DA 2011 Modeling contact friction and joint friction in dynamic robotic simulation using the principle of maximum dissipation. In Algorithmic foundations of robotics IX, vol. 68 (eds Hsu D Isler V Latombe J-C Lin MC). Springer Tracts in Advanced Robotics, pp. 249–266. Berlin, Germany: Springer.

[11] Smith B Kaufman DM Vouga E Tamstorf R Grinspun E 2012 Reflections on simultaneous impact. ACM Trans. Graph. 31, 106 (doi:10.1145/2185520.2185602)

[12] Gilardi G Sharf I 2002 Literature survey of contact dynamics modelling. Mech. Mach. Theory 37, 1213–1239. (doi:10.1016/S0094-114X(02)00045-9)

[13] Khulief YA 2013 Modeling of impact in multibody systems: an overview. ASME J. Comput. Nonlinear Dyn. 8, 021012 (doi:10.1115/1.4006202)

[14] Yin K Loken K van de Panne M 2007 SIMBICON: simple biped locomotion control. ACM Trans. Graph. 26, 105 (doi:10.1145/1276377.1276509)

[15] Wang JM Hamner SR Delp SL Koltun V 2012 Optimizing locomotion controllers using biologically-based actuators and objectives. ACM Trans. Graph. 31, 25 (doi:10.1145/2185520.2185521) 26251560 10.1145/2185520.2185521 PMC4523558

[16] Mordatch I Todorov E Popović Z 2012 Discovery of complex behaviors through contact-invariant optimization. ACM Trans. Graph. 31, 43 (doi:10.1145/2185520.2185539)

[17] Sherman MA Seth A Delp SL 2011 Simbody: multibody dynamics for biomedical research. Proc. IUTAM 2, 241–261. (doi:10.1016/j.piutam.2011.04.023) 10.1016/j.piutam.2011.04.023 PMC4390141 25866705

[18] Djerassi S 2009 Collision with friction; Part B: Poisson's and Stronge's hypotheses. Multibody Syst. Dyn. 21, 55–70. (doi:10.1007/s11044-008-9127-1)

[19] Djerassi S 2010 Reply by the author to Stronge, W.J. Multibody Syst. Dyn. 24, 129–131. (doi:10.1007/s11044-010-9201-3)

[20] Lankarani HM 2000 A Poisson-based formulation for frictional impact analysis of multibody mechanical systems with open or closed kinematic chains. ASME J. Mech. Des. 122, 489–497. (doi:10.1115/1.1319160)

[21] Darboux G 1880 Étude géométrique sur les percussions et le choc des corps. Bull. Sci. Math. Astronom. 4, 126–160.

[22] Keller JB 1986 Impact with friction. ASME J. Appl. Mech. 53, 1–4. (doi:10.1115/1.3171712)

[23] Mirtich B Canny J 1995 Impulse-based simulation of rigid bodies. In Proc. 1995 Symp. on Interactive 3D Graphics, Monterey, CA, 9–12 April, pp. 181–188.

[24] Routh EJ 1905 Dynamics of a system of rigid bodies, 7th edn New York, NY: Macmillan and Co.

[25] Ahmed S Lankarani HM Pereira MFOS 1999 Frictional impact analysis in open-loop multibody mechanical systems. ASME J. Mech. Des. 121, 119–127. (doi:10.1115/1.2829412)

[26] Tasora A Anitescu M 2009 A fast NCP solver for large rigid-body problems with contacts, friction, and joints. In Multibody dynamics, vol. 12 (ed. Bottasso CL). Computational Methods in Applied Sciences, pp. 45–55. Amsterdam, The Netherlands: Springer.

[27] Stewart DE Trinkle JC 1996 An implicit time-stepping scheme for rigid body dynamics with inelastic collisions and Coulomb friction. Int. J. Numer. Methods Eng. 39, 2673–2691. (doi:10.1002/(SICI)1097-0207(19960815)39:15<2673::AID-NME972>3.0.CO;2-I)

[28] Jain A 2013 Minimal coordinates formulation of contact dynamics. In Proc. the 2013 ECCOMAS Thematic Conf. on Multibody Dynamics, Zagreb, Croatia, 1–4 July, pp. 163–180.

[29] Stronge WJ 1991 Unraveling paradoxical theories for rigid body collisions. ASME J. Appl. Mech. 58, 1049–1055. (doi:10.1115/1.2897681)

[30] Goldsmith W 2001 Impact: the theory and physical behaviour of colliding solids. Mineola, NY: Dover.

[31] Hunt KH Crossley FRE 1975 Coefficient of restitution interpreted as damping in vibroimpact. ASME J. Appl. Mech. 42, 440–445. (doi:10.1115/1.3423596)

[32] Quinn DD 2004 Finite duration impacts with external forces. ASME J. Appl. Mech. 72, 778–784. (doi:10.1115/1.1875552)

[33] Pfeiffer F 2003 The idea of complementarity in multibody dynamics. Arch. Appl. Mech. 72, 807–816. (doi:10.1007/s00419-002-0256-3)

[34] Brockley CA Davis HR 1968 The time-dependence of static friction. ASME J. Tribol. 90, 35–41. (doi:10.1115/1.3601558)

[35] Khulief YA 1986 Restitution and friction in impact analysis of multibody systems executing plane motion. In Proc. the 1986 ASME Design Engineering Technical Conference, Columbus, OH, 5–8 October, pp. 86-DET-50.

[36] Cottle RW Pang J-S Stone RE 1992 The linear complementarity problem. New York, NY: Academic Press.

[37] Ivanov AP 1995 On multiple impact. J. Appl. Math. Mech. 59, 887–902. (doi:10.1016/0021-8928(95)00122-0)

[38] Bower AF 2010 Applied mechanics of solids. Boca Raton, FL: CRC Press.

[39] Golub GH Van Loan CF 1996 Matrix computations, 3rd edn Baltimore, MD: The Johns Hopkins University Press.

[40] Wojtyra M Fraczek J 2013 Comparison of selected methods of handling redundant constraints in multibody systems simulations. ASME J. Comput. Nonlinear Dyn. 8, 021007 (doi:10.1115/1.4006958)

[41] Mirtich B 1998 Rigid body contact: collision detection to force computation. Technical Report TR-98-01. Mitsubishi Electric Research Laboratory.

[42] Glocker C 2014 Energetic consistency conditions for standard impacts. Part II: Poisson-type inequality impact laws. Multibody Syst. Dyn. 32, 445–509. (doi:10.1007/s11044-013-9387-2)

[43] Coste C Falcon E Fauve S 1997 Solitary waves in a chain of beads under Hertz contact. Phys. Rev. E 56, 6104–6117. (doi:10.1103/PhysRevE.56.6104)

[44] Liu C Zhao Z Brogliato B 2008 Frictionless multiple impacts in multibody systems. I. Theoretical framework. Proc. R. Soc. A 464, 3193–3211. (doi:10.1098/rspa.2008.0078)

[45] Stronge WJ 2004 Impact mechanics. Cambridge, UK: Cambridge University Press.

[46] Ceanga V Hurmuzlu Y 2001 A new look at an old problem: Newton's cradle. ASME J. Appl. Mech. 68, 575–583. (doi:10.1115/1.1344902)

[47] Luenberger DG Ye Y 2008 Linear and nonlinear programming, 3rd edn New York, NY: Springer.

[48] Johnson KL 1985 Contact mechanics. Cambridge, UK: Cambridge University Press.

[49] Armstrong-Hélouvry B 1991 Control of machines with friction. Norwell, MA: Kluwer.

[50] Hairer E Nørsett SP Wanner G 2008 Solving ordinary differential equations I: nonstiff problems, 3rd edn Berlin, Germany: Springer.

[51] Todorov E 2010 Implicit nonlinear complementarity: a new approach to contact dynamics. In Proc. the 2010 IEEE Int. Conf. on Robotics and Automation (ICRA), Anchorage, AK, 3–7 May, pp. 2322–2329.

[52] Schindler T Nguyen B Trinkle J 2011 Understanding the difference between prox and complementarity formulations for simulation of systems with contact. In Proc. the 2011 IEEE/RSJ Int. Conf. on Intelligent Robots and Systems (IROS), San Francisco, CA, 25–30 September, pp. 1433–1438.

