# Supplementary material for: Making a meaningful impact: modelling simultaneous frictional collisions in spatial multibody systems
Source: Proc Math Phys Eng Sci. 2015 May 8;471(2177):20140859. doi: 10.1098/rspa.2014.0859 (PMC4984984; doi:10.1098/rspa.2014.0859)
Supplement: Appendix [file rspa20140859supp1.pdf]

Appendix

The PLUS impact event handler is summarized in Algorithms 1 and 2, below. Parameters used to generate the simulations described in Section 4 are given in Table A1.

Algorithm 1 (Inner loop) Compute the incremental impulse that advances the impact process to the end of the current interval such that the complementarity condition (5) is respected by all compressing points, and friction cone constraints (6) are satisfied for compressing and expanding points.

Given: The set of proximal points (P), their current tangential velocities ($v_{xy}^{(\mathrm{start})}$), and the set of points that were compressing in the previous round (C_{prev})
Find: The impulse applied in the current impact interval (π)
  Let $E \leftarrow C_{\mathrm{prev}}$ be the set of expanding points
  Let $C \leftarrow P \setminus E$ be the set of compressing points
  Let $S \leftarrow \left\{ p_k \in P : \left\| v_{xy[k]}^{(\mathrm{start})} \right\| \geq v_{\mathrm{transition}} \right\}$ be the set of sliding points
  Let $R \leftarrow P \setminus S$ be the set of rolling points
  loop
    Assemble linear system $B\pi = w$ analogous to (10) and (11)
    Compute $\pi \leftarrow B^{+}w$ using the Moore–Penrose pseudoinverse
    if all constraints are satisfied **then**
      Compute step length α_k from (13) and (14) for each sliding point k
      Let $\alpha \leftarrow \min(\alpha_k)$ be the interval step length
      return $\alpha\pi$
    end if
    Let $p_\ell \leftarrow p \in P$ be the proximal point associated with the largest constraint violation
    if $p_\ell \in R$ **then**
      $R \leftarrow R \setminus p_\ell$
    else
      $C \leftarrow C \setminus p_\ell$
    end if
  end loop

Algorithm 2 (Outer loop) Compute the impulse that approximates the forces that would be applied by a compliant contact model during a collision involving one or more points.

Given: The current velocities of all proximal points (v)
Find: The impulse applied over the duration of the collision ($\pi^{(\text{total})}$)

Initialize $\pi^{(\text{total})} \leftarrow 0$
Let $\pi_z^{(\text{acc})} \leftarrow 0$ be the expansion impulse accumulated in the current round
repeat
  Compute the coefficient of restitution e_k from Figure 2 for each proximal point k
  Let $\pi_z^{(\text{exp})} \leftarrow \pi_z^{(\text{acc})}$ be the expansion impulse to be applied this round
  $\pi_z^{(\text{acc})} \leftarrow 0$
  repeat
    Let $\pi^{(\text{step})}$ be the incremental impulse returned by **Algorithm 1**
    $\pi^{(\text{total})} \leftarrow \pi^{(\text{total})} + \pi^{(\text{step})}$
    $\pi_z^{(\text{exp})} \leftarrow (1-\alpha)\, \pi_z^{(\text{exp})}$
    $\pi_z^{(\text{acc})} \leftarrow \pi_z^{(\text{acc})} + e\pi_z^{(\text{comp})}$
    $v \leftarrow v + A\pi^{(\text{step})}$
  until the current round is complete (i.e., $\alpha = 1$)
until $v_{z[k]} > 0$ for all proximal points k and $\pi_z^{(\text{acc})} = 0$

Table A1: Parameters for the single-point impact scenario of Example 1, shown in Figure 7. The capture velocity, plastic deformation velocity, and minimum coefficient of friction are illustrated in Figure 2; the sliding-to-rolling transition velocity and maximum sliding direction change are defined in Figures 4 and 5, respectively. A unique minimum coefficient of restitution is used with each coefficient of friction.

	Parameter	Value
PLUS impact model	Capture velocity, v_{capture}	0.01 m/s
	Plastic deformation velocity, v_{plastic}	0.1 m/s
	Minimum coefficient of restitution, $e_{\min}$	0.392, 0.387, or 0.373
	Sliding-to-rolling transition velocity, $v_{\text{transition}}$	0.1 m/s
	Maximum sliding direction change, $\theta_{\max}$	0.01 rad
Compliant contact model	Stiffness, E	10 GPa
	Dissipation coefficient, c	0.26 s/m
Physical system	Mass	2.0 kg
	Inertia	$\text{diag}\,(0.167, 0.133, 0.0867)$ kg m^2
	Dimensions	0.4 m $\times$ 0.6 m $\times$ 0.8 m
	Sphere radii	0.1 m
Impact scenario	Pre-impact velocity, $v^{(\text{start})}$	$[-5, 0, -5.74]^{\text{T}}$ m/s
	Coefficient of friction, μ	0.125, 0.225, or 0.325
